# Natural Killer Cells Induce Eosinophil Activation and Apoptosis

**DOI:** 10.1371/journal.pone.0094492

**Published:** 2014-04-11

**Authors:** Ali Awad, Hanane Yassine, Mathieu Barrier, Han Vorng, Philippe Marquillies, Anne Tsicopoulos, Catherine Duez

**Affiliations:** 1 Pulmonary Immunity, Institut National de la Santé Et de la Recherche Médicale, Lille, France; 2 Institut Pasteur de Lille, Center for Infection and Immunity of Lille, Lille, France; 3 CNRS UMR 8204, Lille, France; 4 Univ Lille Nord de France, Lille, France; 5 Clinique des Maladies Respiratoires et Centre Hospitalier Régional et Universitaire de Lille, Lille, France; Centre de Recherche Public de la Santé (CRP-Santé), Luxembourg

## Abstract

Eosinophils are potent inflammatory cells with numerous immune functions, including antigen presentation and exacerbation of inflammatory responses through their capacity to release a range of largely preformed cytokines and lipid mediators. Thus, timely regulation of eosinophil activation and apoptosis is crucial to develop beneficial immune response and to avoid tissue damage and induce resolution of inflammation. Natural Killer (NK) cells have been reported to influence innate and adaptive immune responses by multiple mechanisms including cytotoxicity against other immune cells. In this study, we analyzed the effect of the interaction between NK cells and eosinophils. Co-culture experiments revealed that human NK cells could trigger autologous eosinophil activation, as shown by up-regulation of CD69 and down-regulation of CD62L, as well as degranulation, evidenced by increased CD63 surface expression, secretion of eosinophil cationic protein (ECP) and eosinophil derived neurotoxin (EDN). Moreover, NK cells significantly and dose dependently increased eosinophil apoptosis as shown by annexin V and propidium iodide (PI) staining. Direct contact was necessary for eosinophil degranulation and apoptosis. Increased expression of phosphorylated extracellular signal-regulated kinase (ERK) in cocultured eosinophils and inhibition of eosinophil CD63 expression by pharmacologic inhibitors suggest that MAPK and PI3K pathways are involved in NK cell-induced eosinophil degranulation. Finally, we showed that NK cells increased reactive oxygen species (ROS) expression by eosinophils in co-culture and that mitochondrial inhibitors (rotenone and antimycin) partially diminished NK cell-induced eosinophil apoptosis, suggesting the implication of mitochondrial ROS in NK cell-induced eosinophil apoptosis. Pan-caspase inhibitor (ZVAD-FMK) only slightly decreased eosinophil apoptosis in coculture. Altogether, our results suggest that NK cells regulate eosinophil functions by inducing their activation and their apoptosis.

## Introduction

Eosinophils are multifunctional leukocytes implicated in the pathogenesis of numerous inflammatory processes including parasitic helminth, bacterial and viral infections, tissue injury, tumor immunity, and allergic diseases. Among various hematopoietic factors, IL-5 potently and specifically stimulates eosinophil production and survival [1]. Eosinophils have been shown to possess the ability to perform numerous immune functions, including antigen presentation and exacerbation of inflammatory responses through their capacity to release a range of largely preformed cytokines and lipid mediators [2]. For example, eosinophils can serve as major effector cells inducing tissue damage and dysfunction by releasing an array of cytotoxic granule cationic proteins: Major Basic Protein (MBP), Eosinophil Cationic Protein (ECP) and Eosinophil derived Neurotoxin (EDN) [3]. CD63 translocation and enhanced cell surface expression is associated with this release of mediators [4]. Timely regulation of eosinophil activation and apoptosis is crucial to develop beneficial immune response and to avoid tissue damage and induce resolution of inflammation.

Human Natural Killer cells (NK) are large granular lymphocytes discovered more than 30 years ago, defined by the absence of CD3 and the presence of CD56 on their surface. NK cells constitute approximately 10% to 15% of the total blood lymphocytes and are found in several tissues, including the bone marrow, spleen, liver, omentum, intestine, peritoneal cavity, placenta and lung [5], [6]. They can play a cytotoxic role against stressed, transformed or infected cells by integrating several signals transduced by various activating and inhibitory surface receptors without prior sensitization [7]. In humans, activating receptors include NKp46, NKp30, NKp44 (collectively termed Natural Cytotoxicity receptors, NCR), NKG2D [8], the leucocyte adhesion molecule DNAM-1 (CD226) [9], whereas NKp80 and 2B4 (CD244) [8] are generally considered as co-receptors since their triggering function is dependent on the simultaneous engagement of major activating receptors. LFA-1 (a heterodimer of CD11a/CD18) is required for lysis by NK cells and is sufficient to induce activation signals in NK cells [10]. The main pathway of NK-cell-mediated cytolysis is dependent on perforin and granzymes [11]; however, other mechanisms of target-cell lysis induction have been described, including the role of FAS-L (CD178) and tumor necrosis factor-related apoptosis-inducing ligand (TRAIL)-dependent receptors [12], [13].

NK cells are known to have immunoregulatory effects on immune cells, such as T cells, B cells, dendritic cells, monocytes and neutrophils through cell-cell contact and secretion of various soluble products [14], [15], [16], [17], [18]. For example, they were shown to edit the immune response through induction of lysis or maturation of dendritic cells, a key cell in T lymphocyte polarization [19], [20]. Few studies have evaluated the potential interactions between NK cells and eosinophils, and have provided contradictory results. NK cells were shown to up- or down-regulate allergic eosinophilic inflammation. In murine models of asthma, depletion of NK cells prior to allergen challenge, using anti-NK1.1 or anti-AsialoGM-1 antibodies decreased eosinophilia in bronchoalveolar lavage fluid [21], [22]. However depletion of NK cells after allergen challenge delayed clearance of airway eosinophils and antigen-specific CD4^+^T lymphocytes through NKG2D [23]. In humans, a positive correlation between the eosinophil percentage and NK cell number and activation was observed in peripheral blood from severe asthmatic patients [24]. Supernatants of NK cells from patients with allergic rhinitis were shown to induce *in vitro* recruitment of eosinophils through IL-8 [25]. In contrast, NK cells interacted with autologous eosinophils to promote their apoptosis [24].

The current study was designed to characterize activating or inhibitory effects of NK cells on eosinophils *in vitro* and to investigate the molecular events involved in this scenario. We show that NK cells in co-culture with autologous eosinophils rapidly induce dose-dependent eosinophil degranulation and apoptosis whatever the allergic status of the subjects. Both effects involve cell-cell contact. NK cell-induced eosinophil degranulation depends upon mitogen-activated protein kinase (MAPK) and PI3K pathways, whereas their apoptosis seems to involve mitochondrial ROS.

## Materials and Methods

### Ethic Statement

The study was approved by the Comité consultatif de protection des personnes dans la recherche biomédicale de Lille (CP 04/45). All donors signed an informed consent form.

### Isolation of Peripheral Blood Mononuclear Cells and NK Cell Purification

Blood from adult donors was obtained either through the Etablissement français du Sang in Lille or from allergic or nonallergic volunteers. Human Peripheral Blood Mononuclear Cells (PBMC) were separated from heparinized blood after removal of platelets and plasma (centrifugation 1000 rpm, 12 min, 18°C), dilution of blood in RPMI 1640 (Invitrogen, GIBCO) supplemented with Ticarpen (200 μg/ml) and centrifugation (1800 rpm, 25 min, 18°C) on Ficoll-Paque plus (GE Healthcare, Bio-science AB). After washings in RPMI 1640 (centrifugation 2000 rpm, 10 min, 10°C), PBMC were counted on a Thoma Cell. NK cells were negatively selected as recommended by the manufacturer (StemCell Technologies, Vancouver, BC, Canada V5Z 1B3), after incubation with an antibody cocktail for the depletion of cells expressing the following surface antigens: CD3, CD4, CD14, CD19, CD20, CD36, CD66b, CD123, HLA-DR, glycophorin A, for 10 min at room temperature. This step was followed by incubation for 5 min at room temperature with magnetic beads. NK cells were obtained after incubation for 2.5 min in Easysep magnet and counted on a Thoma Cell with trypan blue to assess their viability and were then resuspended at 1×10^6^ living cells/ml in complete RPMI (RPMI+10% Fetal Calf Serum (FCS) +2 mM glutamine+200 μg/ml Ticarpen). The purity of NK cell fraction (CD3^−^ CD56^+^) was more than 95%, as assessed by flow cytometry (FACScalibur, Becton-Dickinson, California USA) using FITC-conjugated anti-CD3 and PE-conjugated anti-CD56 antibodies (Becton-Dickinson and Beckman Coulter, Fullerton, California).

### Purification of Eosinophils

After the Ficoll step described above, red blood cells from pellets were lysed after two successive incubations (10 mM KHCO_3,_ 155 mM NH4Cl, 0.1 mM EDTA) of 15 and 10 min on ice. Eosinophils were negatively selected by magnetic separation as recommended by the supplier (StemCell Technologies). After 10 min incubation with a cocktail of antibodies for the depletion of cells expressing the following antigens: CD2, CD3, CD14, CD16, CD19, CD20, CD36, CD56, CD123, glycophorin A, granulocytes were incubated for 10 min at room temperature with magnetic beads. Eosinophils were obtained after two successive incubations of 10 min in the Easysep magnet. Living Eosinophils were counted on the Thoma Cell after trypan bleu exclusion and then resuspended at 1×10^6^ living cells/ml in complete RPMI. Purity of eosinophils checked on a slide stained with May-Grünwald Giemsa (Reagent RAL, Martillac, France) was always >95%.

### Co-culture of NK Cells and Eosinophils

After purification, the two cell types were cultured in a 96 round bottom wells (Costar, Corning Incorporated, NY, USA) at different ratio in complete RPMI in the presence of interleukin-5 (20 ng/ml) (R&D Systems, Minneapolis, MN, USA): NK cells alone (shown as ratio 1∶0), eosinophils alone (shown as ratio 0∶1) and NK cells: eosinophils at the ratios 1∶1; 5∶1; 10∶1. Cultures were performed for 3 or 12 hours as indicated in the figures.

### Treatment of Cells

To inhibit the cell contact, NK cells and eosinophils were co-cultured on both sides of Transwell permeable supports 3 μm (Corning Incorporated-Life Sciences). NK cells were also incubated for 1 h 30 in the presence of paraformaldehyde (PFA) 4% and then washed 3 times before incubation with eosinophils. To analyze the role of activating NK cell receptors and co-receptors or death receptors in the modulation of eosinophil function, NK cells were preincubated with different antibodies: anti-FasL (clone NOK1, neutralizing concentrations 10 to 40 μg/ml), anti-NKp46 (clone 9E2/Nkp46, neutralizing concentrations 10 and 20 μg/ml), anti-DNAM-1 (clone DX11, neutralizing concentration 10 μg/ml), anti-LFA-1 (clone HI111, neutralizing concentration 10 μg/ml), anti-CD30L (clone 116614, neutralizing concentrations 5 and 10 μg/ml), mouse IgG1 control (BD Pharmigen), anti-TRAIL (clone 124723, neutralizing concentrations 1 to 40 μg/ml), anti-NKG2D (clone 149810, neutralizing concentrations 10 to 40 μg/ml) (R&D Systems), anti-NKp30 (clone P30–15, neutralizing concentrations 10 to 40 μg/ml) (Biolegend, San Diego, California, USA) and anti-2B4 (clone C1.7, neutralizing concentration 20 μg/ml) (Immunotech, Marseille, France) antibodies. Eosinophils were also preincubated with anti-CD54 (clone HCD54, neutralizing concentration 10 μg/ml) and anti-CD40 (clone 5C3, neutralizing concentrations 1 to 10 μg/ml) antibodies (Biolegend) or mouse IgG1 control antibody (BD Pharmigen). The role of IFN-γ (neutralizing concentration 10 μg/ml) and TNF-α (clone MAB1, neutralizing concentrations 1 to 10 μg/ml) was studied after addition of neutralizing antibodies against each cytokine (R&D system and Biolegend, respectively) or the corresponding control antibodies. To evaluate perforin-mediated cytotoxicity, NK cells were preincubated with Concanamycin A (CMA) (Sigma-Aldrich) 50 nM for 2 h followed by 3 washes before culture with eosinophils. Controls were performed using the corresponding quantities of DMSO used as diluent. To identify signaling pathways involved in their activation, eosinophils were preincubated for 5 min at 37°C, in the presence or absence of inhibitors for P38MAPK, ERK, JNK and PI3K: SB 203580 1 μM, PD 98059 25 μM, SP 600125 20 μM and LY 294002 10 μM, respectively. NK cells were added to the culture without washes. To characterize apoptotic pathways, pan caspase inhibitor (ZVAD-FMK) 50 μM (Calbiochem-Merck, Germany) or inhibitors of mitochondrial electron transport: rotenone 25 μM and antimycin A 45 μM (Sigma-Aldrich, St. Louis, MO) were incubated with eosinophils for 30 min at 37°C before co-culture with NK cells.

### ECP and EDN Measurements

ECP and EDN levels were measured in co-culture supernatants (Medical & Biological Laboratories CO, Japan). Briefly, supernatants or standards were incubated with anti-human ECP or EDN monoclonal antibody for 60 minutes at room temperature. After washings, peroxidase anti-human ECP or EDN polyclonal antibodies was added into the microwells and incubated for 60 minutes at room temperature. After washings, the peroxidase substrate was added for 10 minutes at room temperature. The optical density was read at 450 nm using a microplate reader (Multiskan Ascent, Thermo electron Corporation, WI, USA). The sensitivity of ECP and EDN assays is 0.125 ng/ml and 0.62 ng/ml, respectively.

### Measurement of Apoptosis and Vitality by Flow Cytometry

**Figure 1:**
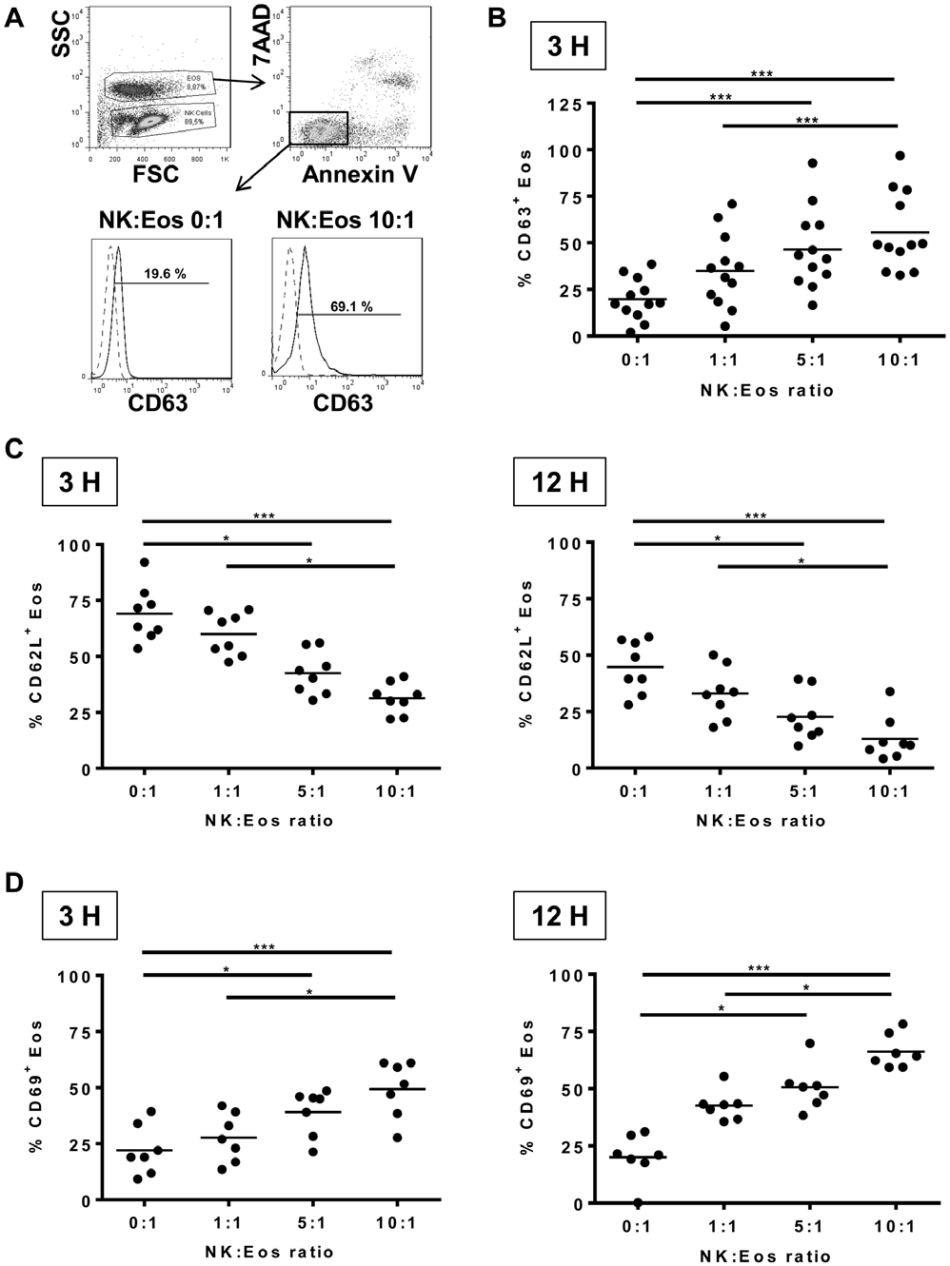
Activation of Eosinophils by NK cells. Autologous NK cells and eosinophils were cultured at the ratio NK cells: eosinophils (NK: Eos ratio) 0∶1 (i.e. eosinophils alone), 1∶1, 5∶1 and 10∶1 in the presence of interleukin-5 for 3 hours and 12 hours. (A) Gating strategy: live eosinophils were identified firstly on the basis of their size difference (FSC) and granularity (SSC) and secondly as Annexin V^−^ 7AAD^−^ cells. CD63 expression (black histogram) is shown on a representative experiment for the ratio NK cells: eosinophils (NK: Eos ratio) 0∶1 (i.e. eosinophils alone) and 10∶1. Isotypic control antibody is represented in dashed grey. Eosinophils were stained for CD63 (B), CD62L (C) and CD69 (D) expression. Results are shown for each donor and mean is indicated with the black line. One-way Anova (Friedman) tests were performed, followed by Dunn’s post tests. *p<0.05; ***p<0.001.

After 3 and 12 hours of co-culture, cells were centrifuged (1500 rpm, 5 min, 4°C) and washed twice in sterile PBS and resuspended in Annexin V Binding Buffer. Annexin V-FITC/Propidium Iodide (PI) staining was performed using 5 μL/test of anti-Annexin V antibody and 5 μL/test of PI as recommended by the manufacturer (BD Pharmingen, CA, USA). The analysis was performed on flow cytometer (FACScalibur piloted by CellQuest Software, Becton-Dickinson). NK cells and eosinophils were identified on the basis of their size difference (FSC) and granularity (SSC) as shown in figure 1. Dead cells were defined as Annexin V^+^ cells, including Annexin V^+^ PI^−^ early apoptotic cells and Annexin V^+^ PI^+^ late apoptotic or dead cells, using FlowJo 7.6.5 software.

### Measurement of Reactive Oxygen Species (ROS) Production by Flow Cytometry

Cells were resuspended at 1×10^6^/ml PBS and labeled with dihydroethidium (Hydroethidine, HE, 1 μM) (Molecular Probes, lifeTechnologies, Grand Island, NY,USA), a substance that is oxidized by ROS to become ethidium and to emit red fluorescence [26]. After 15 min incubation, cells were washed in PBS and analyzed on flow cytometer (FACScalibur).

### Detection of Cell Surface Markers and Intracellular ERK and Phosphorylated-ERK (pERK) by Flow Cytometry

After 3 or 12 hours of co-culture, cells were recovered, centrifuged and resuspended with PBS-SVF 2% for staining of cell surface markers. Cells were incubated at 4°C for 30 min in a V-bottom plate (100 μL/well) (Costar, Corning Incorporated) with antigen specific mouse monoclonal antibodies coupled to a flurochrome or with the corresponding control antibody: anti-CD69-FITC and anti-CD62L-APC (BD Pharmingen) to evaluate activation and anti-CD63-FITC and anti-CD107a-FITC to measure degranulation for eosinophils and NK cells respectively (BD Pharmingen). The expression of the different markers was analyzed on live cells defined as AnnexinV^–^-PE 7-aminoactinomycin D (7AAD)^−^ (BD Pharmingen). After two washes with PBS-SVF2%, cells were resuspended for reading on flow cytometer (FACScalibur). The results were expressed as the percentage of labeled living cells after subtraction of the percentage obtained with the corresponding control antibody.

For ERK and pERK identification, cells were recovered after 3 hours of co-culture and stained with AnnexinV antibody (BD Pharmingen). After fixation with 4% paraformaldehyde (PFA) and permeabilization with 90% methanol, cells were stained with anti-ERK, anti-pERK or the appropriate control antibody according to the manufacturer protocol (Cell Signaling Technology, MA, USA). Staining was evaluated on AnnexinV^−^ cells using flow cytometer (FACScalibur).

### Statistical Analysis

When appropriate, D’Agostino and Pearson normality test was performed and values were expressed as mean ± standard error of the mean (SEM). Comparisons were performed with the Wilcoxon test or the one- or two-way ANOVA tests followed by Bonferroni, Tukey, Dunn or Sidak post tests using the software Graph Pad Prism 6 (Graph Pad software, USA) as mentioned in figure legends. The results were considered significant for p<0.05.

## Results

### NK Cells Induce Eosinophil Activation and Degranulation

**Figure 2:**
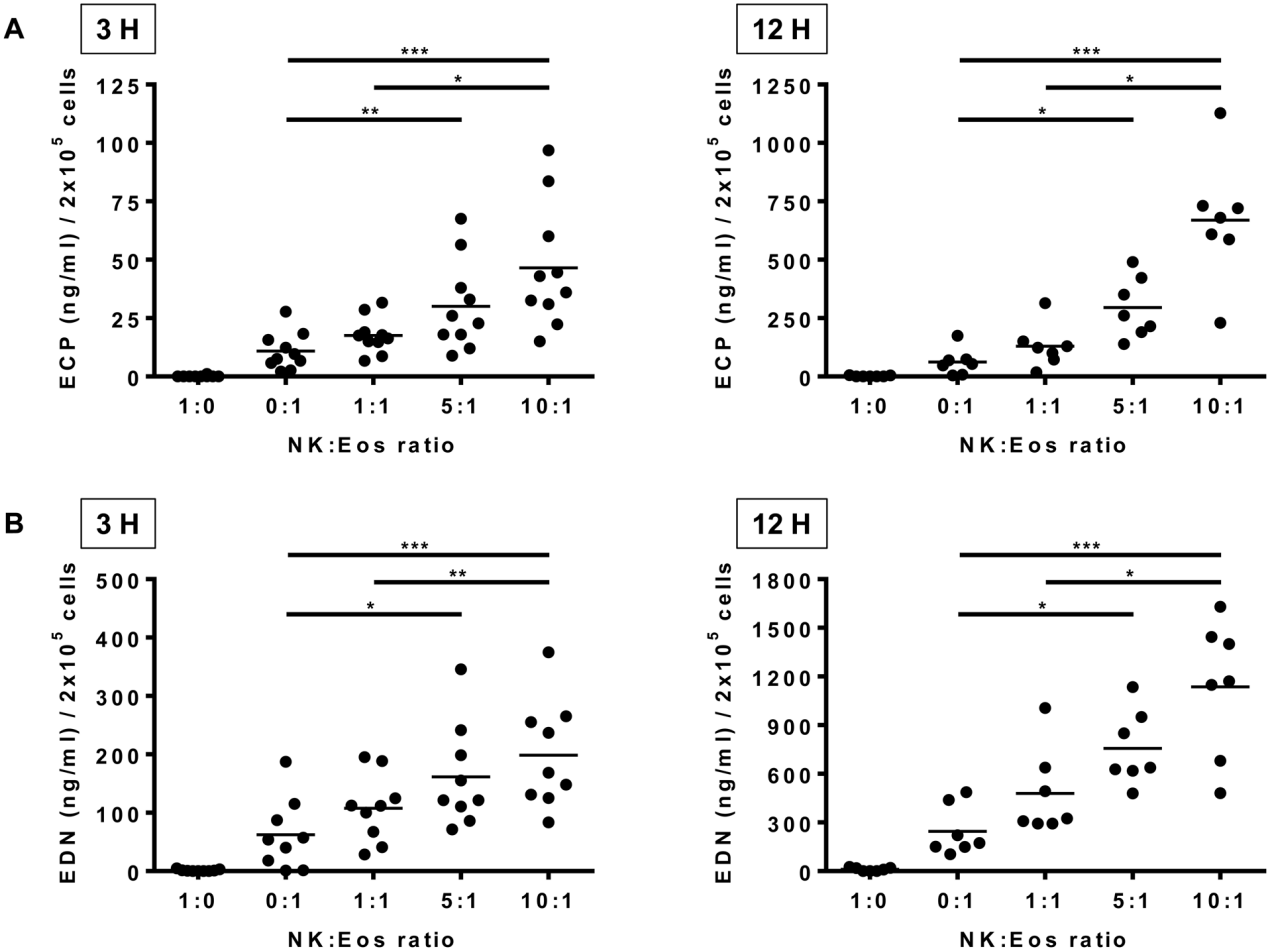
NK cells induce eosinophil release of ECP and EDN. Levels of eosinophil cationic protein (ECP) (A) and eosinophil derived neurotoxin (EDN) (B) were measured in co-culture supernatants after 3 and 12 hours for the NK:Eos ratios 1∶0 (i.e. NK cells alone), 0∶1 (i.e. eosinophils alone); 1∶1; 5∶1; 10∶1. Results are expressed as molecule quantity in ng/ml for 2×10^5^ cells for each donor and mean is indicated with the black line. One-way Anova (Friedman) tests were performed, followed by Dunn’s post tests. *p<0.05; **p<0.01; ***p<0.001.

The effect of human NK cells on eosinophil activation was analyzed *in vitro* after purification of both cell types from fresh blood of the same donor. Because cytokine priming occurs *in vivo* in a variety of allergic, parasitic and other hypereosinophilic disorders [27], and because eosinophils die rapidly without survival factors, cultures were performed in the presence of IL-5. Eosinophil activation was evaluated after 3 and 12 h at different ratios of NK cells:eosinophils (0∶1 i.e. eosinophils alone, 1∶1, 5∶1 and 10∶1) by the measurement of cell surface expression of CD62L and the early activation marker CD69 (figure 1C–D). In order to evaluate if eosinophil degranulation comes along with activation, CD63 surface expression [4] was measured after 3 hours of co-culture. Live eosinophils were defined as annexin V^−^ 7AAD^−^ cells with high side scatter parameter (figure 1A). Results showed that eosinophils co-cultured with autologous NK cells significantly and dose-dependently decreased CD62L (figure 1C) and increased CD69 (figure 1D) expression at 3 and 12 h, which is consistent with eosinophil activation. Similarly, eosinophils expressed higher CD63 surface expression when co-cultured with increasing numbers of NK cells (figure 1B), suggesting degranulation. Eosinophil degranulation was confirmed by the detection of ECP (figure 2A) and EDN (figure 2B) in co-culture supernatants, detectable both at 3 and 12 hours. No ECP/EDN levels were detected in supernatant culture of NK cells alone. ECP and EDN release increased with quantities of NK cells and time.

### NK Cells Induce Eosinophil Apoptosis

**Figure 3:**
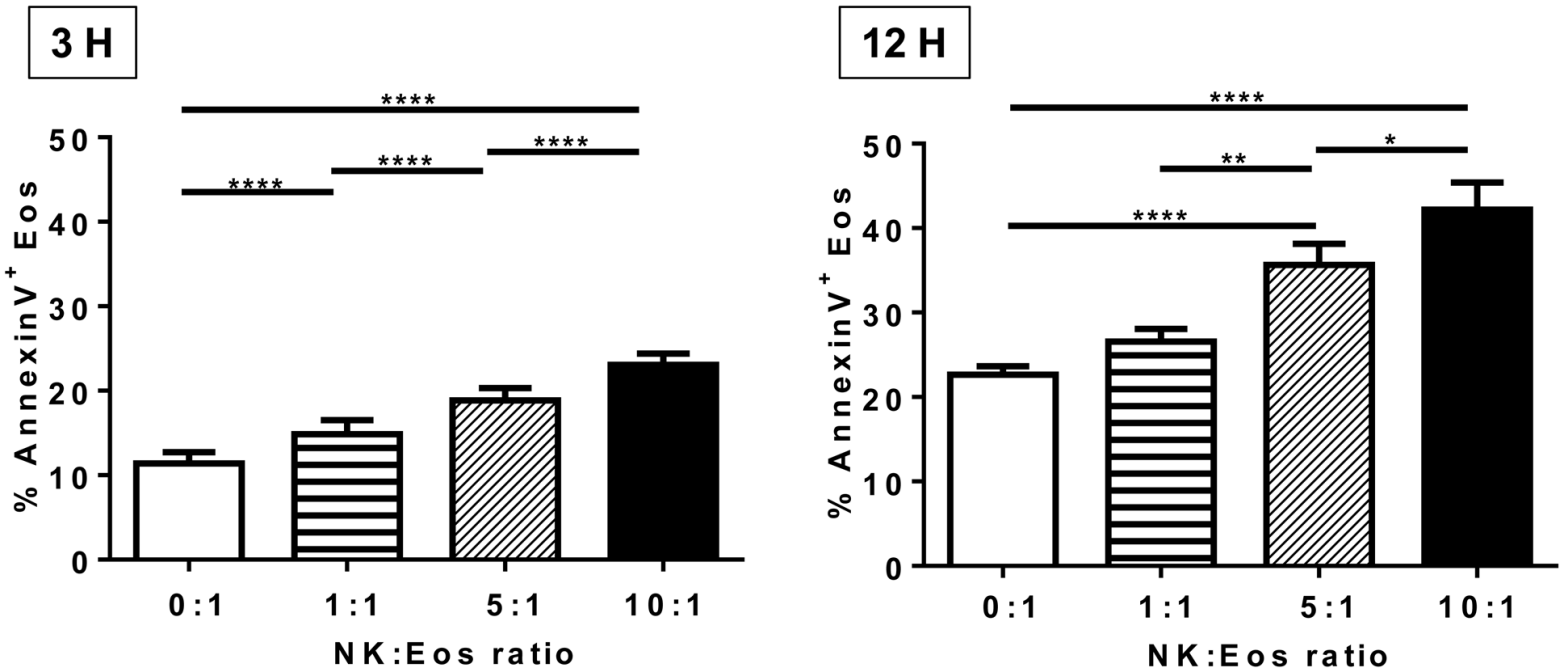
NK cells induce eosinophil apoptosis. After purification, NK cells and eosinophils were cultured in the presence of IL-5 for 3 and 12 h at different NK:Eos ratios: 0∶1 (i.e. eosinophils alone); 1∶1; 5∶1; 10∶1. Eosinophils were identified on the basis of their size difference (FSC) and granularity (SSC). Apoptosis and death of eosinophils was evaluated by Annexin/PI labeling. Dead eosinophils were defined as AnnexinV^+^ cells. Results are expressed as mean percentage of AnnexinV^+^ cells amongt the eosinophil population ± SEM. n = 16 (3 hours) or 18 (12 hours). Gaussian distributions were verified using D’Agostino and Pearson test, and One-way Anova tests were performed, followed by Bonferroni’s post tests. *p<0.05; **p<0.01; ***p<0.001.

We evaluated whether resting NK cells were also able to modify eosinophil apoptosis in the same culture conditions. Eosinophil apoptosis was detected by annexin V and propidium iodide (PI) staining after 3 and 12 h incubation with different ratios of NK cells. The culture with NK cells significantly and dose dependently increased eosinophil apoptosis at 3 and 12 hours (figure 3). Eosinophil apoptosis also increased with time. Because delayed eosinophil apoptosis has been observed in several eosinophil-associated diseases including allergic asthma and rhinitis [28], we compared the effect of NK cells on the apoptosis of autologous eosinophil from allergic (asthma or rhinitis) and non allergic donors. Eosinophils were similarly sensitive to NK cells whatever the allergic status, and no significant difference except the apoptosis levels of eosinophils alone was observed (data not shown). Therefore data from allergic and non allergic donors are both included in the figures. Finally eosinophils did not modify NK cell apoptosis (data not shown).

### Cellular Interaction and Soluble Mediators are Involved in NK Cell-induced Eosinophil Degranulation and Apoptosis

**Figure 4:**
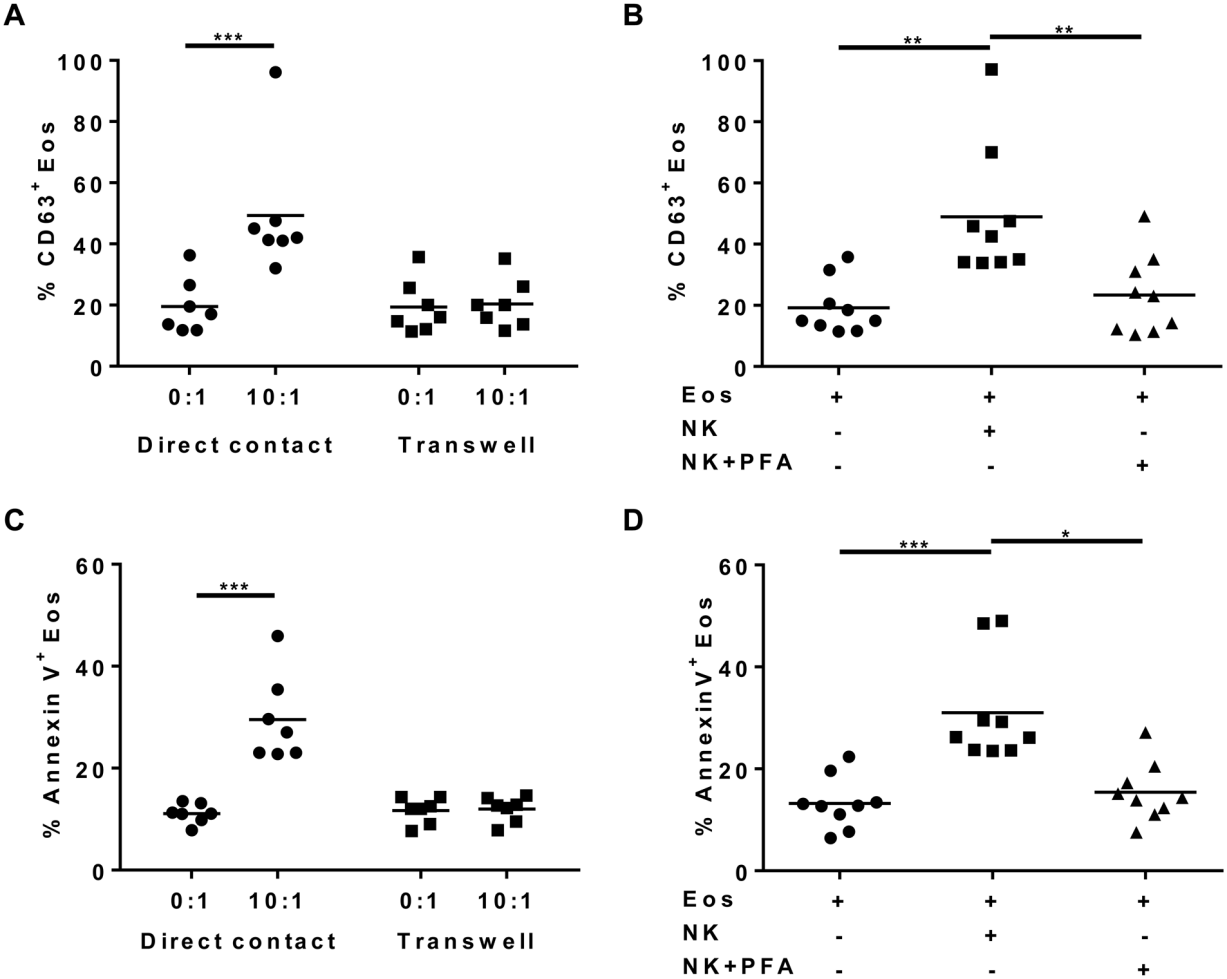
Eosinophil degranulation and apoptosis are both abrogated after PFA-induced fixation of NK cells and separation of cell types by transwell. After purification, NK cells and eosinophils were cultured in the presence of IL-5 for 3 hours at the NK:Eos ratios: 0∶1 (i.e. eosinophils alone) and 10∶1. (A) Percentage of CD63 positive eosinophils cultured with NK cells at the 0∶1 (i.e. eosinophils alone) or 10∶1 NK:Eos ratios in the same culture well (direct contact) or in Transwell (separated with 3 μm filter). (B) Percentage of CD63 positive eosinophils cultured alone, with live NK cells or with PFA-fixed NK cells for the 10∶1 NK:Eos ratio. (C) Percentage of AnnexinV^+^ eosinophils cultured with NK cells at the 0∶1 (i.e. eosinophils alone) or 10∶1 NK:Eos ratios in the same culture well (direct contact) or in Transwell (separated with 3 μm filter). (D) Percentage of AnnexinV^+^ eosinophils cultured alone, with live NK cells or with PFA-fixed NK cells for the 10∶1 NK:Eos ratio. Results are shown for each donor and mean is indicated with the black line. One-way Anova (Friedman) tests were performed, followed by Dunn’s post tests. *p<0.05; **p<0.01; ***p<0.001.

To analyze if cell contacts or soluble mediators were involved in eosinophil activation, degranulation and apoptosis, eosinophils were cultured alone or with NK cells (10∶1 ratio), either separated by a permeable membrane (Transwell) or fixed with paraformaldehyde (PFA), respectively. The presence of a Transwell membrane between cells completely abrogated the increased surface expression of CD63 on eosinophils compared to direct contact, suggesting the involvement of surface molecule interactions (Figure 4A). Similarly, NK cells separated from eosinophils by the membrane did not modify the apoptosis of eosinophils after a co-culture of 3 h (Figure 4C) or 12 h (data not shown), suggesting that a direct contact is necessary to induce eosinophil apoptosis. We tested the involvement of adhesion molecules (DNAM-1, LFA-1 and CD54), NK cell cytotoxic activating receptors (NKp30, NKp46, NKG2D), co-receptor (2B4), members of the TNF family ligands (FasL, TRAIL, CD40L) or membrane TNF-α by the use of blocking antibodies in co-culture experiments. None of the neutralizing antibody impaired CD63 increased expression and apoptosis induced by NK cells (data not shown), suggesting the involvement of molecules different from the ones classically implicated in the interaction of NK cells with their target cells.

In order to avoid the participation of soluble mediators produced by NK cells (such as perforins, granzymes, IFN-γ and TNF-α) on eosinophil degranulation and apoptosis, NK cells were fixed with paraformaldehyde (PFA) prior to co-culture. Indeed, human peripheral blood NK cells fixed with PFA were previously shown to retain cytotoxic properties attributable to the constitutive expression on their surface of cytotoxic molecules like FasL or membrane TNF-α [29], suggesting that NK cell surface molecules remain functional, although we cannot exclude that PFA may affect the dynamics of interactions between some receptors and their ligands. Fixation of NK cells significantly decreased the percentage of CD63 positive eosinophils (Figure 4B), suggesting that soluble factors may be involved. To analyze the role of IFN-γ, co-cultures were performed in the presence of neutralizing antibodies. No modifications in the percentage of CD63 positive eosinophils were seen (data not shown), suggesting that IFN-γ is not implicated in NK cell-induced eosinophil degranulation. Moreover, no IL-6, IL-8, IL-10, TNF-α, CCL3 and lipoxin A4 were detected by ELISA in coculture supernatants (data not shown). Similarly, NK cell fixation significantly decreased eosinophil apoptosis (Figure 4D), suggesting the involvement of soluble factors in this function. However, concanamycin A, which inhibits perforin-based cytotoxic activity [30], did not inhibit eosinophil apoptosis after 3 h and 12 h of co-culture (data not shown). We also observed that NK cell in co-culture with eosinophils did not modify their CD107a surface expression (data not shown), a marker of NK cell degranulation [31]. These results suggest that NK cells did not induce eosinophil apoptosis through granule exocytosis as a cytotoxic pathway.

### MAP Kinases Participate in Eosinophil Degranulation Induced by NK Cells

**Figure 5:**
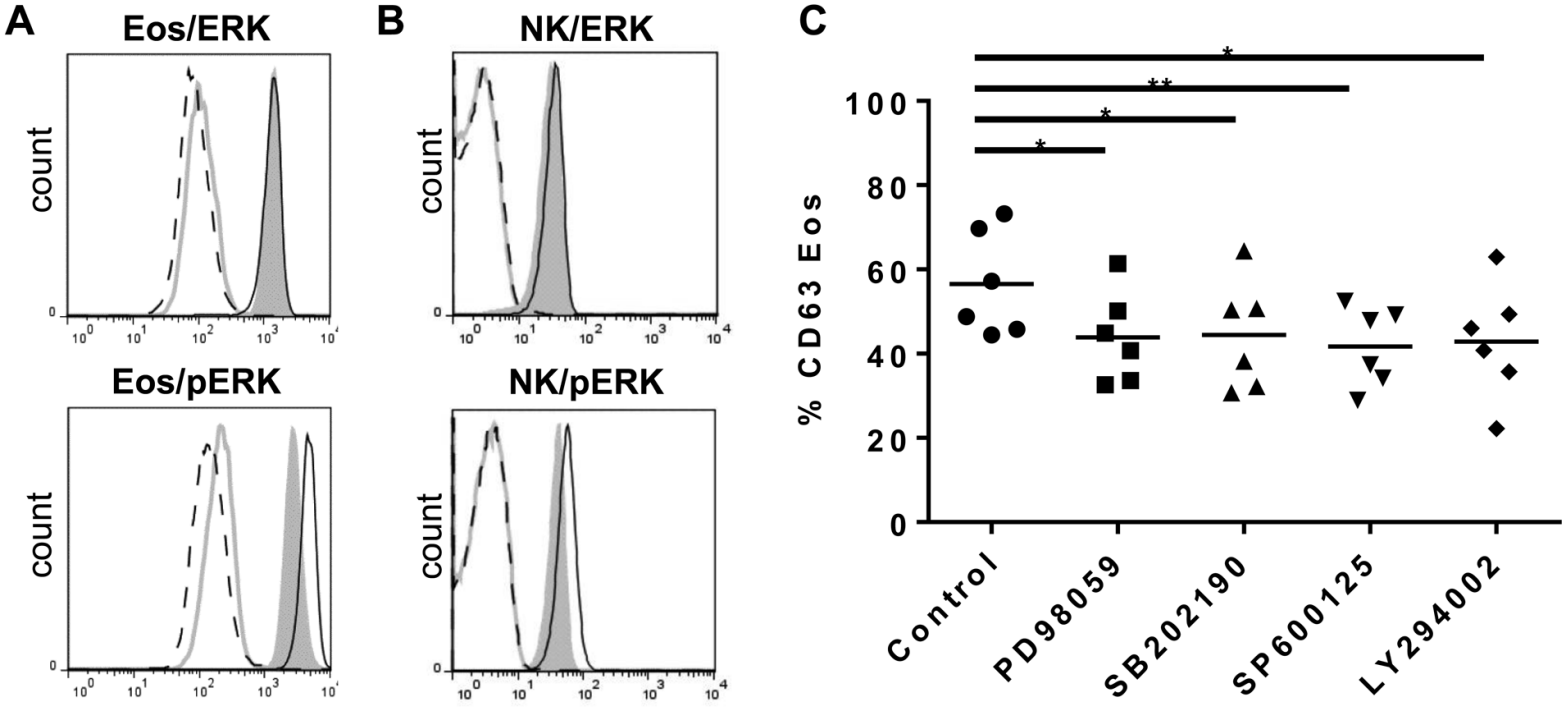
MAPK and PI3K pathways are involved in eosinophil degranulation. AnnexinV^−^ eosinophils (A) and NK cells (B), cultured alone or together at the 10∶1 NK:Eos ratio, were stained with anti-ERK or anti-phosphorylated ERK (pERK) antibodies. Eosinophils and NK cells were identified on the basis of their size difference (FSC) and granularity (SSC). Live eosinophils were defined as AnnexinV^−^ cells. Gray full lines and black dotted lines represent control antibody staining for each cell type alone or in co-culture, respectively. Gray plain histograms and black full lines represent staining with antibody of interest for each cell type alone or in co-culture, respectively. One representative experiment out of three is shown. (C) Eosinophils were pre-incubated with DMSO as control or with inhibitors of signaling pathways for 5 min at 37°C: PD 98059 25 μM an inhibitor of ERK pathway, SB 203580 1 μM an inhibitor of P38 MAPK, SP 600125 20 μM an inhibitor of JNK pathway and LY 294002 10 μM an inhibitor of PI3K pathway for the NK:Eos ratio 10∶1. Results show percentage of CD63^+^ eosinophils for each donor and mean is indicated with the black line. One-way Anova (Friedman) tests were performed, followed by Dunn’s post tests. *p<0.05, **p<0.01.

The mitogen-activated protein kinase (MAPK) pathway (including extracellular signal-regulated kinase (ERK), c-Jun Nterminal kinase (JNK), and p38 MAPK) as well as the Akt cascade involving the production of phosphatidylinositol 3,4,5 triphosphates (PtdIns(3,4,5)P3) by phosphoinositide 3-kinase (PI3K) have been shown to play a role in eosinophil degranulation induced by various stimuli [32], [33]. Eosinophils in co-culture with NK cells exhibited increased phosphorylated ERK (pERK) compared to eosinophils alone (figure 5A). Similarly, NK cells from the co-culture increased their expression of pERK compared to NK cells alone (figure 5B), suggesting that NK cells are activated in the presence of eosinophils. To study the importance of MAP kinases and PI3K pathways in NK cell-induced eosinophil degranulation, we added SB202190 (a p38 inhibitor), PD98059 (a MAP/ERK kinase inhibitor), SP600125 (an inhibitor of the three JNK enzymes) or LY294002 (a PI3K inhibitor) to co-cultures of NK cells and eosinophils. Corresponding quantities of DMSO were used for control conditions. All inhibitors significantly decreased the percentage of CD63 positive eosinophils (figure 5C), suggesting that these pathways are, at least partly, involved in NK cell-induced eosinophil degranulation. No significant effect was measured on NK cell-induced eosinophil apoptosis. Moreover, none of the inhibitors decreased spontaneous eosinophil degranulation and apoptosis (data not shown).

### Depletion of the Mitochondrial Respiratory Chain Prevents NK Cell-induced Eosinophil Apoptosis

**Figure 6:**
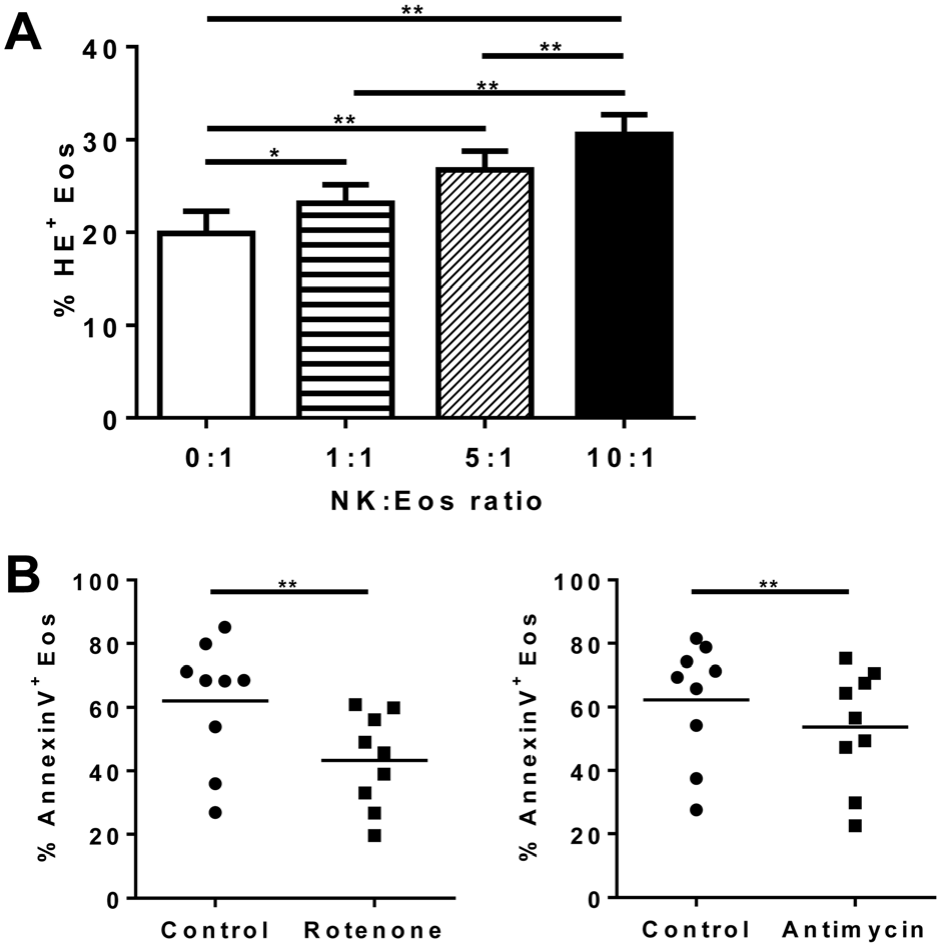
NK cell-induced eosinophil apoptosis involves mitochondrial reactive oxygen species (ROS). (A) After purification, NK cells and eosinophils were cultured in the presence of IL-5 for 12 hours at different NK:Eos ratios: 0∶1 (i.e. eosinophils alone); 1∶1; 5∶1; 10∶1. Eosinophils were identified on the basis of their size difference (FSC) and granularity (SSC). ROS staining was achieved using dihydroethidium (HE). Results are expressed as mean percentage of HE^+^ cells amongt the eosinophil population ± SEM. n = 12. Gaussian distributions were verified using D’Agostino and Pearson test, and One-way Anova test was performed, followed by Bonferroni’s post test. *p<0.05; **p<0.01. (B) Eosinophils were incubated with inhibitors of mitochondrial electron transport: rotenone and antimycin for 30 min at 37°C before 12 h co-culture with NK cells at the NK:Eos ratio of 10∶1. Results for each donor are shown and mean is indicated with the black line. Wilcoxon tests were performed. *p<0.05.

We followed superoxyde production in eosinophils by staining with the superoxide sensitive probe dihydroethidium (HE). NK cells dose-dependently increased the percentage of HE-positive eosinophils (figure 6A), suggesting that eosinophils generated higher levels of reactive oxygen species (ROS) following co-culture with NK cells. Rotenone suppresses the release of superoxide from mitochondria by binding to the ubiquinol binding site in respiratory complex 1, thereby blocking the flow of electrons from complex I to complex III [34], whereas antimycin prevents electron flow in the mitochondrial respiratory chain, acting on complex III [35]. Optimal non toxic inhibitory concentration of rotenone and antimycin were first determined on eosinophils at 25 μM and 45 μM, respectively (data not shown). Both mitochondrial inhibitors partially but significantly blocked NK cell-induced eosinophil apoptosis (figure 6B), suggesting the involvement of mitochondrial ROS. Rotenone also significantly decreased spontaneous apoptosis of eosinophils cultured in the presence of IL-5 only (data not shown).

To evaluate if caspase activation may signal for NK cell-induced eosinophil apoptosis, co-cultures were performed in the presence of general caspase inhibitor (ZVAD-FMK). ZVAD-FMK slightly but significantly (Wilcoxon test, p<0.05) decreased the apoptosis of eosinophils in co-culture with NK cells at the ratio 10∶1 (median for ZVAD: 63.45%; interquartile range: 54.18%, 76.10%; median for control: 70%; interquartile range: 59.63%, 78.70%), but not for eosinophils alone. This result implicates a minor role of caspases.

## Discussion

In this study, we showed that resting NK cells induce dose-dependent *in vitro* activation of autologous eosinophils after 3 and 12 hours of co-culture. Activated eosinophils degranulated as showed by the increased surface expression of CD63 on live eosinophils and ECP and EDN release in culture supernatants. CD63 has been involved in eosinophil activation and piecemeal degranulation of eosinophils [4]. Activation and cytotoxic protein release increased with time. When separated from NK cells by a permeable filter, eosinophils did not degranulate anymore, suggesting the need for cell interactions. None of the cell surface molecule tested was found to be involved. In particular, NKp30 which plays a key role in NK cell interactions with dendritic cell [19], [20], was not implicated. Neutralization of other activating receptors like NKp46 and NKG2D failed to inhibit NK cell-induced eosinophil activation. 2B4 functions as a co-receptor in human NK cell activation [36]. Its ligand CD48 is widely expressed by hematopoietic cells, and more particularly by eosinophils. Cross-linking CD48 on human eosinophils was demonstrated to trigger release of eosinophil granule proteins [37]. However 2B4/CD48 interaction was not involved in NK cell-induced eosinophil degranulation. Finally, co-cultures were performed in the presence of anti-DNAM-1 antibody. DNAM-1 is expressed by NK cell and interacts with CD112, expressed by eosinophils and involved in eosinophil activation [38], [39]. Anti-DNAM-1 antibody did not inhibit the expression of CD63 on eosinophils. We then co-cultured eosinophils with fixed NK cells, previously shown to allow some cellular interactions while blocking the release of soluble mediators [29]. Eosinophils did not degranulate in this experimental setting, suggesting the involvement of soluble mediators, although we cannot exclude that PFA may affect the dynamics of the interactions between some receptors and their ligands. IFN-γ, which induce eosinophil degranulation [4] did not play a role in NK cell-induced degranulation. Because NK cells may produce high mobility group B1 (HMGB1) [40], and HMGB1 induces eosinophil degranulation (MBP and EPO release) [41], we tested the neutralization of HMGB1 in NK cell/eosinophil co-culture and found no effect. Finally, IL-6, IL-8, IL-10, TNF-α, CCL3 and lipoxinA4 were evaluated in culture supernatants. None of these mediator was detected, suggesting that they were not involved either.

We then investigated the signaling pathway involved in NK cell-induced eosinophil degranulation. Inhibitors for ERK1/2, p38MAPK, JNK and PI3K pathways added to the co-cultures significantly decreased eosinophil degranulation assessed by CD63 surface expression, suggesting their involvement in NK cell-induced eosinophil degranulation. ERK1/2, p38 MAPK, PI3K were shown to play a role in eosinophil degranulation induced by various stimuli [32], [33], but are also implicated in NK functions (cytokine production or cytotoxicity) [42], [43], [44], [45]. pERK was increased in NK cells cocultured with eosinophils. Therefore, we cannot separate the inhibitory action of SB202190, PD98059, SP600125 and LY294002 on eosinophils from the effect on NK cells.

In parallel, we showed that resting NK cells in co-culture with eosinophils induced their apoptosis, with no differences in apoptotic rates in allergic versus non allergic donors. This is in accordance with previously published results, where only NK cells from severe asthmatics display lower NK cell-induced apoptosis, whereas NK cells from mild asthmatics and healthy subjects had similar levels of apoptosis [24]. Kinetic experiments revealed that increased eosinophil apoptosis was detected as early as one minute after co-culture, and paralleled activation/degranulation of live eosinophils (data not shown). This observation suggests that eosinophil apoptosis is directly induced by NK cells and is not naturally occurring as a consequence of eosinophil activation. This is also suggested by the absence of inhibitory effects of MAPK and PI3K inhibitors on eosinophil apoptosis, while they decreased eosinophil degranulation in the presence of NK cells (data not shown). Moreover, inhibitors of NK cell-induced apoptosis (rotenone and antimycin) did not decrease eosinophil activation (data not shown). Finally, no association was found between the percentage of CD63^+^ eosinophils and the percentage of annexinV^+^ eosinophils (spearman correlation, r = 0.2585, p = 0.1121), suggesting that NK cell-induced eosinophil activation and apoptosis are mainly, but not totally, independent.

In the current study, NK cell-induced eosinophil apoptosis was shown to be dependent upon contact by the transwell experiment and on soluble mediators. This is concordant with the formation of the NK-cell immunological synapse, which begins with contact with a target cell and culminates either in the directed delivery of lytic granule contents to lyse the target cell [46], or in the release of soluble mediators to activate immune cells [47]. However, we found none of the cytotoxic receptor (NKp30, NKp46, and NKG2D) or coreceptor (2B4) or adhesion molecule (DNAM-1, LFA-1, and ICAM-1) to be involved. This is concordant with the absence of apoptosis inhibition observed using concanamycin, an inhibitor of perforin-induced degranulation. Similarly to the Barnig et al study [24], we did not evidence the expression of CD107a and CD107b, indicative of NK cell degranulation [31]. FAS-L, TRAIL and membrane TNF-α were not implicated either, as showed by neutralizing antibodies treatment. Our results suggest that eosinophil apoptosis is not induced by NK cell classical cytotoxic functions, in contrast to dendritic cells and neutrophil apoptosis [18], [19]. NK cells induced increased ROS detection in eosinophils, while mitochondrial inhibitors partially inhibited NK cell-induced eosinophil apoptosis. The conjunction of both results suggests the involvement of mitochondrial ROS in NK cell-induced eosinophil apoptosis. Eosinophil apoptosis may be dependent or independent of caspases, depending on the stimuli. For example, Siglec-8–mediated cell death in IL-5–activated eosinophils is caspase independent but involves reactive oxygen species and MEK/ERK activation [48]. In our experiments, a pan-caspase inhibitor only slightly decreased eosinophil apoptosis, suggesting that the apoptosis pathway is mainly caspase independent.

Interestingly, our results showed that resting NK cells *in vitro* modulate eosinophil activation and apoptosis, with no need for other stimuli. Eosinophils may have stimulated NK cells like dendritic cells do [49]. We did not evidence NK cell activation by CD62L and CD69 staining (data not shown) but increased percentage of NK cells expressed pERK when co-cultured with eosinophils. Resting NK cells were previously shown to trigger apoptosis of both eosinophils and neutrophils [24]. Nevertheless, most studies co-cultured activated NK cells with hematopoietic cells. For example, short-term IL-12-activated NK cells induced significant apoptosis in neutrophils [18], whereas NK cells activated with IL-2, IL-15, IL-21, IL-12, or IL-18 during the co-culture decreased their apoptosis [50]. Also, supernatants of IL-15-activated NK cells recruited eosinophils [25]. We suspect a crosstalk between NK cells and eosinophils, leading to NK cell activation sufficient for eosinophil activation and/or apoptosis.

Our *in vitro* results are in agreement with studies in murine models of asthma. Indeed, NK cell-induced eosinophil degranulation may participate to allergic eosinophilia. Depletion of NK cells prior to allergen challenge, using anti NK1.1 or anti-AsialoGM-1 antibodies decreased eosinophilia in bronchoalveolar lavage [21], [22]. In parallel, we showed that NK cells induced eosinophil apoptosis after co-culture. This correlates with delayed clearance of airway eosinophils after depletion of NK cells with anti-AsialoGM-1 antibodies performed after allergen challenge [23]. Therefore, time of depletion may reveal different functions of NK cells: they may up- and down-regulate eosinophilic inflammation by activating eosinophils or inducing their apoptosis, respectively.
